# Fine mapping of a *Phytophthora*-resistance gene *RpsWY* in soybean (*Glycine max* L.) by high-throughput genome-wide sequencing

**DOI:** 10.1007/s00122-017-2869-5

**Published:** 2017-02-28

**Authors:** Yanbo Cheng, Qibin Ma, Hailong Ren, Qiuju Xia, Enliang Song, Zhiyuan Tan, Shuxian Li, Gengyun Zhang, Hai Nian

**Affiliations:** 1 The State Key Laboratory for Conservation and Utilization of Subtropical Agro-bioresources, South China Agricultural University, Guangzhou, 510642 Guangdong People’s Republic of China; 2 The Key Laboratory of Plant Molecular Breeding of Guangdong Province, College of Agriculture, South China Agricultural University, Guangzhou, 510642 Guangdong People’s Republic of China; 3 Beijing Genomics Institute (BGI)-Shenzhen, Shenzhen, 518086 People’s Republic of China; 4 Agricultural Research Service, Crop Genetics Research Unit, United States Department of Agriculture, Stoneville, MS 38776 USA

## Abstract

**Key message:**

Using a combination of phenotypic screening, genetic and statistical analyses, and high-throughput genome-wide sequencing, we have finely mapped a dominant *Phytophthora* resistance gene in soybean cultivar Wayao.

**Abstract:**

*Phytophthora* root rot (PRR) caused by *Phytophthora sojae* is one of the most important soil-borne diseases in many soybean-production regions in the world. Identification of resistant gene(s) and incorporating them into elite varieties are an effective way for breeding to prevent soybean from being harmed by this disease. Two soybean populations of 191 F_2_ individuals and 196 F_7:8_ recombinant inbred lines (RILs) were developed to map *Rps* gene by crossing a susceptible cultivar Huachun 2 with the resistant cultivar Wayao. Genetic analysis of the F_2_ population indicated that PRR resistance in Wayao was controlled by a single dominant gene, temporarily named *RpsWY*, which was mapped on chromosome 3. A high-density genetic linkage bin map was constructed using 3469 recombination bins of the RILs to explore the candidate genes by the high-throughput genome-wide sequencing. The results of genotypic analysis showed that the *RpsWY* gene was located in bin 401 between 4466230 and 4502773 bp on chromosome 3 through line 71 and 100 of the RILs. Four predicted genes (*Glyma03g04350, Glyma03g04360, Glyma03g04370*, and *Glyma03g04380*) were found at the narrowed region of 36.5 kb in bin 401. These results suggest that the high-throughput genome-wide resequencing is an effective method to fine map PRR candidate genes.

**Electronic supplementary material:**

The online version of this article (doi:10.1007/s00122-017-2869-5) contains supplementary material, which is available to authorized users.

## Introduction

*Phytophthora* root rot (PRR) caused by *Phytophthora sojae* is a one of the most important soil-borne diseases in many soybean-production regions in the world, which was first noted in America in 1948 (Schmitthenner 1985; Wrather et al. 2001; Tyler 2007). PRR caused global soybean production losses of $10–20 billion (Tyler 2007). Since PRR was first recorded in Heilongjiang province in 1991 (Shen and Su 1991), reports on new races of *Phytophthora sojae* and their virulence diversity have emerged continuously in major production areas of soybean (Zhu et al. 2004; Cui et al. 2010; Zhang et al. 2010; Huang et al. 2016). PRR can reduce soybean yield by 10–40% or a complete yield loss (Li and Ma 1999; Zhang et al. 2013a), indicating that PRR is a serious disease potential threat to soybean production in China (Huang et al. 2016).

Soybean resistance to *P. sojae* is controlled by two mechanisms, partial or complete resistance genes (Sugimoto et al. 2012). The mechanism of partial resistance involves multiple genes and limits damage to the plant (Schmitthenner 1985; Dorrance et al. 2003). The other mechanism is related to a single dominant gene resistance (Hartwig et al. 1968; Burnham et al. 2003a; Fan et al. 2009; Yao et al. 2010; Yu et al. 2010; Sugimoto et al. 2011; Sun et al. 2011, 2014; Wu et al. 2011b; Zhang et al. 2013b), in which *P. sojae* interacts with *Rps* genes in a gene-for-gene system preventing disease development in plants (Schmitthenner 1999).

To date, 22 *Rps* genes/alleles on eight different soybean chromosomes have been identified, among which *Rps1* (five alleles *Rps1a, Rps1b, Rps1c, Rps1d*, and *Rps1k*), *Rps7, Rps9, RpsYu25, RpsYD29*, and *RpsUN1* and an unnamed *Rps* gene in soybean Wascshiroge and E00003 were mapped on the short arm of chromosome 3 (Demirbas et al. 2001; Weng et al. 2001; Gao et al. 2005; Fan et al. 2009; Sugimoto et al. 2011; Sun et al. 2011; Wu et al. 2011a; Lin et al. 2013; Zhang et al. 2013b, 2014). *Rps2* and *RpsUN2* were found on chromosome 16 (Demirbas et al. 2001; Lin et al. 2013). *Rps3* (three alleles *Rps3a, Rps3b* and *Rps3c*) and *RpsSN10* which was linked with *Rps8* were on chromosome 13 (Demirbas et al. 2001; Sandhu et al. 2005; Gordon et al. 2006; Yu et al. 2010). In addition, *Rps4, Rps5, Rps6*, and *RpsJS* were located on chromosome 18 (Demirbas et al. 2001; Sandhu et al. 2004; Sun et al. 2014a, b), and *Rps8, RpsYB30, RpsZS18, RpsSu, Rps10*, and *Rps11* were located on chromosome 8, 19, 2, 10, 17 and 7, respectively (Burnham et al. 2003a; Zhu et al. 2007; Yao et al. 2010; Wu et al. 2011b; Zhang et al. 2013c; Ping et al. 2016).

Partial resistance or field resistance to *P. sojae* is controlled by quantitative trait loci (QTL) and/or several genes. More than 70 QTLs have been found for partial resistance to *P. sojae* in soybean (Burnham et al. 2003b; Weng et al. 2007; Han et al. 2008; Li et al. 2010; Tucker et al. 2010; Wang et al. 2010, 2012a, b; Wu et al. 2011c; Nguyen et al. 2012; Lee et al. 2013a, b, 2014; Sun et al. 2014a, b; Schneider et al. 2016). Fine mapping using RILs constructed with resistant germplasm and susceptible alleles has been broadly used to identify QTLs underlying tolerance to PRR in recent years (Burnham et al. 2003b; Weng et al. 2007; Li et al. 2010; Tucker et al. 2010; Wang et al. 2010, 2012a, b; Wu et al. 2011c; Nguyen et al. 2012; Lee et al. 2013a, b). Some resistant cultivars (Conrad, Hefeng 25, MN0902, PI398841, and etc) were investigated to compare the phenotypes and yield contributions of QTLs for partial resistance to *P. sojae* in soybean (Burnham et al. 2003b; Jia et al. 2008; Li et al. 2010; Lee et al. 2013a). The results from QTLs, gene analysis, association mapping, joint linkage QTL analyses, and genome-wide association mapping indicate that partial resistance to PRR is regulated by a complex QTL-mediated resistance network (Burnham et al. 2003b; Weng et al. 2007; Li et al. 2010; Wang et al. 2010, 2012a, b; Nguyen et al. 2012; Lee et al. 2014; Sun et al. 2014a, b; Schneider et al. 2016).

Recently, single nucleotide polymorphisms (SNPs) have emerged as genetic markers for their high density and relatively even distribution in the plant genomes. With the rapid development of high-throughput sequencing technology, SNP markers have become practical and give good efficiency and accuracy of QTL mapping (Wang et al. 2011; Duan et al. 2013). Some new sequencing technologies for SNP genotyping of soybean genetic map have been developed with the completion of the whole-genome sequencing of soybean cv. Williams 82 (Hyten et al. 2008, 2010; Li et al. 2014; Lin et al. 2014; Qi et al. 2014). A high-density integrated genetic linkage map consisting of 5500 SNP markers for soybean was constructed (Hyten et al. 2008). Li et al. (2014) used specific length amplified fragment sequencing (SLAF-seq) from RILs to construct a high-density soybean genetic map with 5785 SLAFs. A novel ion transporter gene *GmCHX1* was identified using the de novo sequencing and germplasm resequencing approaches (Qi et al. 2014). All these techniques contribute greatly to identify major QTLs or genes in soybean.

In addition, the use of recombination bins which presumably capture all recombination events in the population can provide abundant markers based on dense SNPs for detailed genome-wide trait analysis and serve as a new and effective type of genetic markers for QTL analysis. Huang et al. (2009) constructed a dense genetic map using recombination bins as markers for 150 rice (*Oryza sativa* L.) RILs by the method of whole-genome sequencing for genotyping recombinant populations. Wang et al. (2011) mapped 49 QTLs of rice at high resolution by sequenced-based genotyping using recombinant inbred lines. However, identification of soybean *Rps* genes using soybean RILs population with high-throughput sequencing technology has not been reported.

In this study, we found that the cv. Wayao had broad-spectrum resistance and may carry *Rps* genes or alleles. The objectives of our project were to characterize the inheritance of the *Rps* gene(s) and fine map the candidate gene(s) in the resistant cv. Wayao.

## Materials and methods

### Plant materials

The soybean cv. Wayao and Huachun2 were obtained from the Guangdong Subcenter of National Center for Soybean Improvement, South China Agricultural University. To determine which *Rps* gene or *Rps* gene combination were present in Wayao, we used a differential set of cultivars/genotypes. Each cultivar/genotype carries one known single *Rps* gene shown as: Harlon (*Rps1a*), Harosoy13XX (*Rps1b*), Williams79 (*Rps1c*), PI103091 (*Rps1d*), Williams82 (*Rps1k*), L76-988 (*Rps2*), L83-570 (*Rps3a*), PRX146-36 (*Rps3b*), PRX145-48 (*Rps3c*), L85-2352 (*Rps4*), L85-3059 (*Rps5*), Harosoy62XX (*Rps6*), and Harosoy (*Rps7*). The cv. Williams (*rps*) and Huachun 2 were used as the susceptible checks to indicate successful inoculation. Forty individual seedlings of each cultivar were also injured using the method of injured hypocotyl inoculation (Sun et al. 2011). All the differential hosts for PRR identification were kindly provided by the National Center for Soybean Improvement, Nanjing Agricultural University.

The mapping populations of 191 F_2_ individuals and 196 F_7:8_ RILs were derived from Huachun 2 (P_1_, PRR susceptible to the isolates of Pm14, Pm28, PNJ1, PNJ3, PNJ4, and P6497) × Wayao (P_2_, PRR resistance to the isolates of Pm14, Pm28, PNJ1, and P6497). Two F_1_ seeds from the cross were self-pollinated to produce the population of F_2_ plants. One F_2_ plant containing 191 seeds was used for F_2_ individual population. The 196 F_7:8_ RILs from the other F_2_ plant were developed with the single seed descent method (Brim 1966).

### Preparation of the *P. sojae* isolates and culture medium

Six *P. sojae* isolates (Pm14, Pm28, PNJ1, PNJ3, PNJ4, and P6497), which were provided by Prof. Yuanchao Wang and Han Xing at Nanjing Agricultural University (Wu et al. 2011b) and were used in the phenotype test of disease resistance to PPR. The *P. sojae* isolates were preserved on the slant medium after the processes of strain rejuvenation, separation, and purification (Wu et al. 2011b). The Pm14 strain was used to screen the plant resistance of the F_2_ and RILs population of Huachun 2 × Wayao.

The V8 juice-calcium carbonate medium was prepared using V8 vegetable juice (Campbell Soup Company). After 1.2 g of calcium carbonate was added into 120 ml of V8 vegetable juice, the mixture was centrifuged for 8 min at 25 °C in 4000 rpm. One hundred millilitre supernatant was taken out and put into a volumetric flask to be a constant volume with ultra-pure water and 15 g agar to 1 L. The mixture was then autoclaved at 121 °C for 20 min. Finally, the solid medium was made from the mixture in petri dishes of 9-cm diameter with appropriate thickness of 1.5 mm.

### Evaluation of genetic materials for *Phytophthora* resistance

For inoculation with *P. sojae*, all the seeds of F_2_ population were sown and germinated in a 9-cm diameter plastic bucket filled with vermiculite. To test the phenotypes of the F_2_ population, the isolate Pm14 (virulence formula is *1a, 1b, 1c, 1d, 1k, 2, 3a, 3c, 4, 5, 6, 7*) was used to inoculate the parental cultivars Huachun 2 and Wayao, individual seedlings of F_2_ population using injured hypocotyl inoculation method with slight modification (Sun et al. 2011). Briefly, after sowing 7-day-old seedlings were inoculated, a uniform and thinner wound was cut in soybean cotyledon under section 1–2 cm using single-sided blade sterilized with the outer flame of alcohol lamp. The active edge of the colony from *P. sojae* isolate Pm14 cultured in the incubator at 25 °C for 5 days was cut into about 3 mm^2^ block and then embedded into the wound area with mycelium surface inside. After inoculation, the seedlings were placed in the culture shelf surrounded by plastic film and kept moisture (90% relative humidity) at 25 °C for 24 h with spraying sterilized water, and then transferred to the pure light room at 20–30 °C with a 14-h light and 10-h dark cycle. Reactions of the seedlings were evaluated 5 days after inoculation. Reactions of F_2_ population were recorded as S (susceptible, seedling dead with brown hypocotyls) or R (resistant, seeding alive with no expanding lesion).

To test the phenotypes of RILs population, 12 seeds from every line for each replication were sown and germinated in a 9-cm diameter plastic bucket filled with vermiculite with three replications. During the process of seed germination and seedlings growth, the light time was set to 14 h per day with a photosynthetic active radiation of 80 µmol m^−2^ s^− 1^ and the culturing room temperature of 30 °C, while the dark time was set to 10 h per day with the culturing room temperature of 24 °C. Ten selected individuals from the 12 seedlings for each replication were then further inoculated with the isolate Pm14. The parental cultivars of Huachun 2 and Wayao, individual seedlings of RILs population were evaluated using injured hypocotyl inoculation method (Sun et al. 2011). Reactions of the seedlings were evaluated 5 days after inoculation. Reactions of RILs population were recorded as the percentage of dead seedlings 5 days after inoculation. The RIL families with 70–100% dead seedlings were considered to be homozygous susceptible (S), while the RIL families with 0–30% dead seedlings were considered to be resistance (*R*). One hundred and forty four individual seedlings of Wayao and Huachun2 were used as the resistance and susceptible checks, and forty individual seedlings of Williams were used as the susceptible checks to indicate successful inoculation. All the experiments were performed in a culturing room at the South China Agricultural University.

### F_2_ DNA preparation and pooling for bulk segregation analysis

Leaf samples were taken from the young seedlings of parents and individual plants of the F_2_ population. PCR was conducted according to Sun et al. (2011). The genomic DNA from each individual plant was extracted using CTAB (cetyl trimethylammonium bromide) method with minor modifications (Allen et al. 2006). Ten selected homozygous-resistant and ten homozygous-susceptible F_2_ individuals were used to prepare the DNA pooling for bulk segregation analysis (BSA). Resistant or susceptible bulk of DNA was prepared by pooling equal amounts of 1 μg DNA from each of the selected resistant or susceptible individuals, respectively (Michelmore et al. 1991). The final concentration of each bulk was adjusted to 30 ng/μl for PCR analysis (Michelmore et al. 1991).

### SSR markers and linkage analysis of resistance gene in F_2_

A total of 613 SSR markers uniformly covering the 20 soybean chromosomes were obtained from Soybase (http://soybase.org/) were used to screen DNA polymorphisms among the two parents and resistant and susceptible bulks of DNA. There were 210 SSR markers polymorphic between the two parents. Four SSR markers (Table S1) were polymorphic between resistant and susceptible bulks of DNA (Michelmore et al. 1991). These four polymorphic markers were further used for analyzing the genotypes and the types of the electrophoretic bands of the F_2_ population. The SSR reaction program and system were carried out by the method of SSR analysis outlined by Sugimoto et al. (2008).

A genetic linkage map of the *Rps* genes was constructed using the Mapmaker 3.0b software. Chromosomes were determined with a log-likelihood (LOD) threshold of 3.0. The electrophoresis band types of F_2_ individuals were compared to those of parents based on the results of SSR markers. The bands consistent with those of soybean parent Wayao were marked as A (1), and the bands consistent with those of soybean parent Huachun 2 were marked as B (3), while the heterozygous bands with those of both parents were marked as H (2). The individual of disease-resistant phenotype was denoted by R (1), while the individual of susceptible phenotype was denoted by S (0).

### Genome-wide sequencing and analysis

The high-throughput genome-wide sequencing was performed to construct a high-density genetic map for fine mapping using the 196 F_7:8_ RIL populations at BGI-Shenzhen. The genomic DNA was extracted from the parents and the RILs plants using the method of CTAB (Duan et al. 2013).

Genome-wide SNP development and genotyping for the RIL population were performed using MSG (multiplexed shotgun genotyping) as proposed by Duan et al. (2013). The pair-end read libraries were prepared with bar-coded adapters designed and modified by the standard Illumina adapter design. 1 μl FastDigest Taq I (Thermo scientific Fermentas) was used to digest 1 μg genomic DNA of each sample in a 30 μl reaction for 10 min at 65 °C. 10 μmol unique barcode adapters were then transferred into each well for genomic DNA sample. A ligation reaction with T4 DNA ligase (Enzymatics) were hatched for an hour at 22 °C and followed by a heat inactivation at 65 °C for 20 min. All the 24 ligation products were collected into one tube for different samples, and then the restriction enzyme was inactived with 2 μl chloroform. The 400–600 bp fragments separated on a 2% agarose gel were purified by a QIAquick Gel Extraction Kit. A 10-cycle PCR of 50 μl reaction, including 25 μl Phusion Master Mix, 1 μl of 10 μM common primer and 1 μl index primer, was carried out to amplify all the purified products for each sample (Phusion high-fidelity, Finnzymes). The amplified library purified using a QIAquick PCR Purification Kit was sequenced on a Hiseq 2000 instrument after being quantified on Agilent 2100 Bioanalyzer.

SOAPaligner software (Li et al. 2009a, b) was used to compare sequencing reads of each individual according to the identification tag sequence with soybean reference genome. The input file for realSFS using SAMtools (Li et al. 2009a, b) was gained after format conversion of the results by SOAP comparison. The information for every site of RIL population was identified by realSFS to obtain the genotype of each individual according to those of Huachun 2 and Wayao soybeans for reference. Then, the exchange sites of each individual were determined to get the bin genotype of each individual by the genotypes of the parents and the population. Finally, the genetic linkage fine map was constructed according to the bin genotype of each individual using MSTMap (http://alumni.cs.ucr.edu/~yonghui/mstmap.html) and MapChart softwares (Voorrips 2002).

### QTL mapping

After constructing linkage map, WinQTLCart2.5 was used for QTL analysis. Composite interval mapping (Zeng 1993, 1994) was performed for all traits. A 10 cM window at a walking speed of 1 cM was used in a stepwise forward regression procedure, and 5 markers were used to eliminate inherent background effects among linked multiple QTL. LOD threshold was calculated using 1000 permutations for an experimentalwise error rate of *P* = 0.05. When the positions of QTL affecting a trait overlapped among several locations, they were interpreted to be the same QTL if they fell within a range of 10 cM. Additive effects and phenotypic variance were estimated by QTL (R2) at the highest peaks shown in WinQTLCart2.5 analyses.

## Results

### Phenotype reaction of Wayao to *P. sojae* isolates

To investigate the pathotype of Wayao and Huachun 2, six isolates of *P. sojae* were used to test the PRR reaction on 16 soybean genetic differentials (Table 1). The inoculation results showed that Huachun 2, Williams, L76-988 and PRX145-48 were susceptible to all six isolates (SSSSSS). It appears that Huachun 2 did not contain disease-resistant genes, or did not contain *Rps2* or *Rps3c* resistance genes. PRX146-36 (RSSSSR) and Wayao (RRRSSR) were PRR-resistant cultivars to Pm14 strain, while other cultivars were PRR-susceptible cultivars to Pm14 strain (Table 1). Furthermore, Wayao was also PRR-resistant to Pm28 and PNJ1 strains different from that of PRX146-36. These results suggest that Wayao may contain new *Rps* genes.

**Table 1:** Differential reactions of soybean hosts and cultivars to strains of *P. sojae*

Cultivar	*Rps*	*Phytophthora sojae* strains					
		Pm14	Pm28	PNJ1	PNJ3	PNJ4	P6497
Harlon	*1a*	S	S	R	S	S	R
Harosoy13XX	*1b*	S	S	R	S	S	S
Williams79	*1c*	S	S	R	S	S	R
PI103091	*1d*	S	S	S	S	S	R
Williams82	*1k*	S	S	R	S	S	R
L76-988	*2*	S	S	S	S	S	S
Chapman	*3a*	S	R	R	R	R	R
PRX146-36	*3b*	R	S	S	S	S	R
PRX145-48	*3c*	S	S	S	S	S	S
L85-2352	*4*	S	R	S	R	S	R
L85-3059	*5*	S	S	R	S	R	R
Harosoy62XX	*6*	S	S	S	R	S	R
Harosoy	*7*	S	S	S	S	R	S
Williams	*rps*	S	S	S	S	S	S
Huachun 2		S	S	S	S	S	S
Wayao		R	R	R	S	S	R

### Genetic analysis of resistance to *P. sojae* Pm14

The PRR resistance cultivar Wayao plants showed no symptoms in response to *P. sojae* Pm14 after 5-day inoculation, while the susceptible cultivars Huachun 2 and Williams plants showed severe rot at the inoculation site and all the inoculated plants ultimately died. The segregation ratio was investigated using the individual plants of F_2_ and RILs population derived from the cross of Huachun 2 × Wayao. The F_2_ and RILs fit well with a genotypic Mendelian 3R:1 S ratio and 1R:1S ratio, respectively (${X^{\text{2}}}_{{\text{3}}:{\text{1}}}$ = 0.21 < ${X^{\text{2}}}_{0.0{\text{5}}}$ = 3.84, *P* > 0.05; ${X^{\text{2}}}_{{\text{1}}:{\text{1}}}$ = 0.16 < ${X^{\text{2}}}_{0.0{\text{5}}}$ = 3.84, *P* > 0.05) (Table 2). These results indicate that the resistance gene in Wayao is controlled by a single dominant gene, temporarily designated as *RpsWY*.

**Table 2:** Segregation analysis of resistance to *P. sojae* Pm14 in cross of Huachun 2 × Wayao

Cross or parent	Total no. of plants/lines			Chi squared tests		
	Resistance	Segregation	Susceptibility	Expected ratio	*X* ^2^	*P*
Williams	0	0	40			
P_1_	0	0	144			
P_2_	144	0	0			
F_2_	146	0	45	3:1	0.21	3.84
F_7:8_	102	0	94	1:1	0.16	3.84

### Mapping *RpsWY* gene with SSR markers

**Fig. 1:**
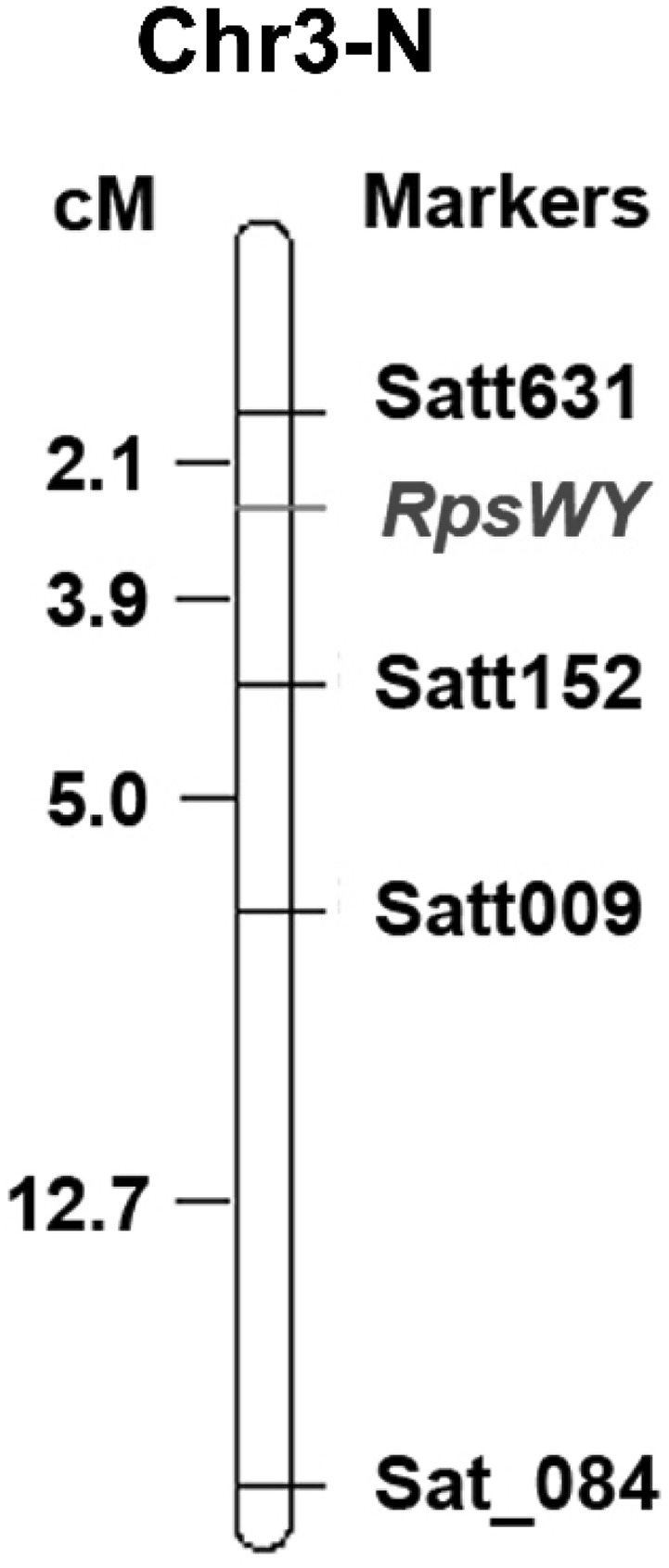
Genetic map of *RpsWY* on chromosome 3. A total of 210 polymorphic SSR markers were obtained from Soybase (http://soybase.org/). The genetic map was constructed using the 191 F_2_ population derived from the cross of Huachun 2 × Wayao using the multipoint analysis of Mapmaker 3.0b software. A linkage group was established in a threshold of LOD score 12.64. Chr3-N: Chromosome 3 of MLG N

Bulk-segregant analyses were used to determine the map location of the unknown *Rps* gene. Four markers (Satt152, Satt631, Satt009, and Sat_084) on chromosome 3 were investigated and showed DNA polymorphic fragments between the DNA pools (Table S1). The results showed that *RpsWY* was linked to these four SSR markers and located between Satt631 and Satt152 at a distance of 2.1 and 3.9 cM, respectively (Fig. 1).

### SNP genotyping

To investigate the SNP markers, the cultivars Wayao and Huachun 2 were resequenced using the Illumina Hiseq2000 platform with sequencing average depths of 3.85× and 7.72×, respectively (Table 3). The statistical index changed according to the average depth of sequencing between Wayao and Huachun 2. The short reads were mapped back to the genome of soybean Williams 82 using SOAP2 (version 2.20). Approximately, 45.80 and 96.38 Mbp reads, and 4.12 and 8.67 Gbp bases of raw data were obtained. The mapped reads of Huachun 2 and Wayao were 41.34 and 83.75 Mbp, respectively. While the mapped bases of each genotype were 3.72 and 7.54 Gbp, respectively, the genome coverage of these two cultivars was 87.50 and 94.63%, respectively (Table 3). A total of 787 882 SNPs were identified between the parents using a strict analysis pipeline (Table S2). However, only 70,000 SNPs were found in the RIL population (Table S2).

**Table 3:** Sequencing of Wayao and Huachun 2 soybeans

Cultivars	Total reads (M)	Total base (G)	Mapped reads (M)	Mapped base (G)	Coverage (%)	Average depth (X)
Huachun2	45.80	4.12	41.34	3.72	87.50	3.85
Wayao	96.38	8.67	83.75	7.54	94.63	7.72

**Fig. 2:**
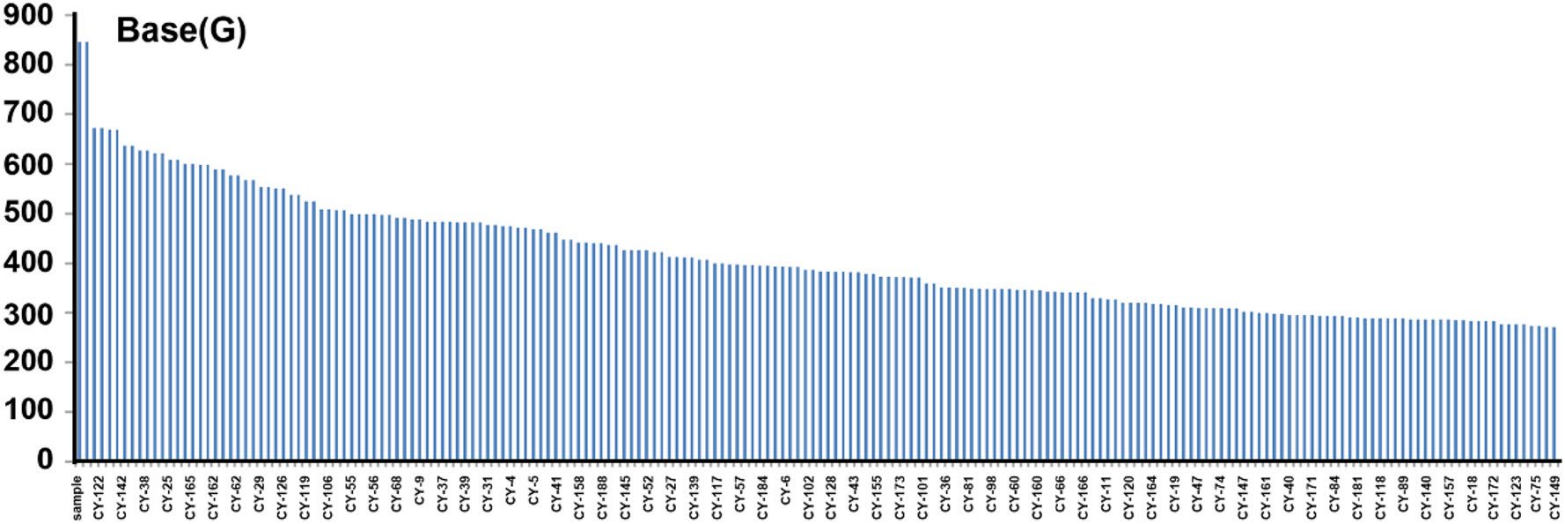
Sequencing data statistics of each individual of RILs population. One single plant was taken from every line of the 196 F_7:8_ RILs population. Horizontal axis shows different *lines* of RIL population, and vertical coordinate shows total numbers of bases (Mbp)

We resequenced 196 lines of the RILs using 0.2× RAD-seq (restriction association site DNA sequence) to get the short reads mapped back to the genome of soybean Williams 82 using SOAP2 (version 2.20). All the individuals from 196 lines of RILs were also resequenced using the Illumina Hiseq 2000 platform with an average sequencing depth of 4.88× and coverage rate of 9.44% (Table S3). The sequencing data showed that the mapped bases of the RILs were 58.70 Gbp with the average bases of 301.03 Mbp. 145 soybean lines had more than 200 Mbp bases, and 183 soybean lines had more than 100 Mbp bases. Only line CY-176 had less than 100 Mbp bases (Fig. 2).

Population SNPs were filtered by the sites which were different between the two parents. The SNPs due to noise were removed manually. A total of 70,000 SNPs or 1 SNP per 5 kb were detected for the RILs. The distribution of SNPs was even throughout the entire genome (Table 4).

**Table 4:** Bin number and Linkage distance of Chromosomes

Chromosome	Number of bins	Linkage distance
Gm01	177	262.2
Gm02	189	264.7
Gm03	184	209.9
Gm04	108	182.2
Gm05	169	240.1
Gm06	212	238.9
Gm07	152	190.5
Gm08	197	252.9
Gm09	214	234.9
Gm10	145	211.2
Gm11	177	209.0
Gm12	128	174.2
Gm13	216	250.8
Gm14	132	184.2
Gm15	143	176.6
Gm16	201	235.9
Gm17	174	182.0
Gm18	199	263.2
Gm19	159	173.9
Gm20	193	206.0
Total	3469	3855.5

### Construction of high-density genetic map

**Fig. 3:**
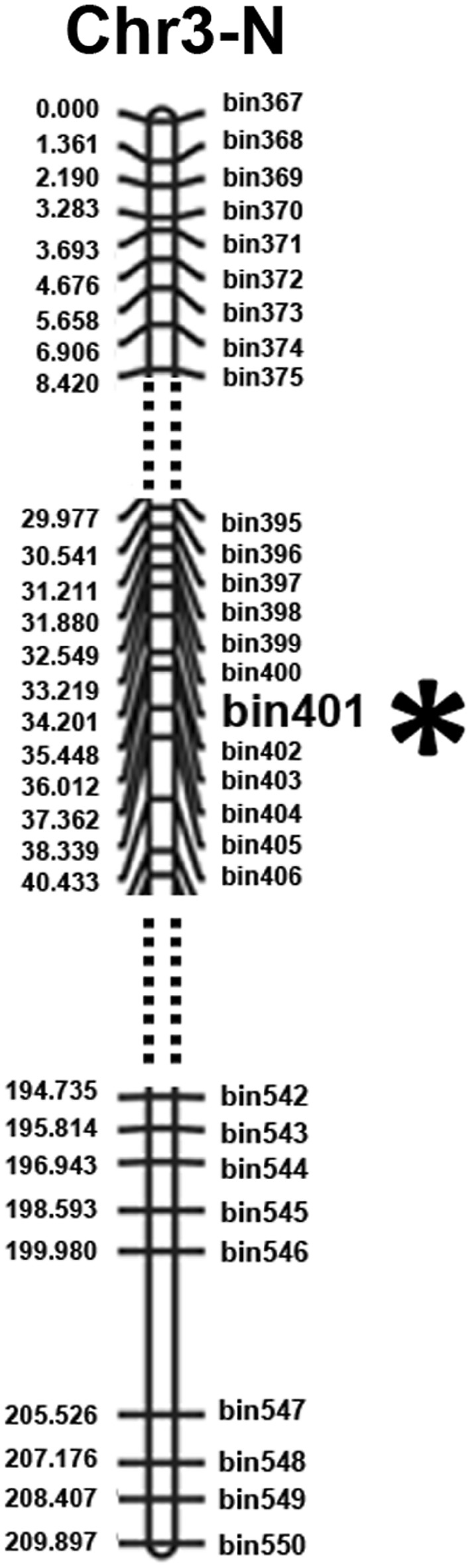
Genetic map of soybean chromosomes. The virtual point line represents the truncated segment of the chromosome 3. The *asterisk* indicates the location of bin401

Using the sliding window approach, we chose 15 SNPs for a window as the genetic bin marker distance to identify the genotype for each window and exchange sites for each individual when it slides an SNP every time (Huang et al. 2009; Duan et al. 2013; Wang et al. 2016). Bin information was generated using the genotype for each individual (Huang et al. 2009). After determining the recombination breakpoints for each individual, a total of 3469 bins were detected for the 196 RILs (Table 4). A high-density map was constructed using the bins as markers (Fig. 3; Fig. S1, S2).

**Fig. 4:**
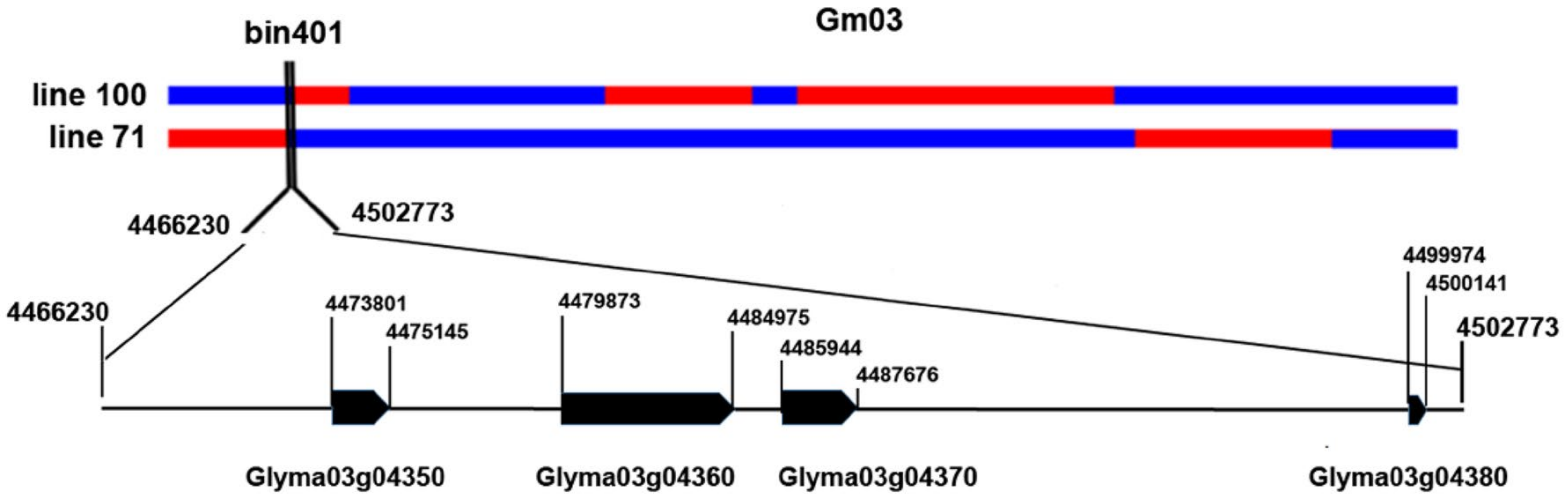
Fine mapping of *RpsWY*. Recombinant Line71 and Line100 were from the RILs population. *Blue blocks* designates Wayao genotype on chromosome 3, and *red blocks* designates Huachun 2 genotype on chromosome 3. Line71 and Line100 were resistant to *P. sojae* Pm14. Four candidate *Rps* genes of *Glyma03g04350, Glyma03g04360, Glyma03g04370*, and *Glyma03g04380* were located at the region of recombinant bin401. The position information of candidate genes was from the website of soybase: http://soybase.org/. (Color figure online)

Using CIM (composite interval mapping method) with WinQTLCart for *Rps* gene localization, *RpsWY* gene was amplified at the site of bin401 on chromosome 3 in the high-density linkage map (Fig. 3). The *RpsWY* gene was located in the region of 4,466,230–4,502,773 bp through recombinant monoclonal line 71 and line 100 which were resistant to *P. sojae* Pm14 strain (Figs. S3 and S4). Four candidate *Rps* genes of *Glyma03g04350, Glyma03g04360, Glyma03g04370*, and *Glyma03g04380* were located at the region (Fig. 4). The results of bioinformatic analysis showed that *Glyma03g04350* encodes a predicted pentatricopeptide repeat-containing protein possibly involved in RNA editing (Kotera et al. 2005), *Glyma03g04360* encodes a predicted transposase/serine/threonine protein phosphatase, while *Glyma03g04370* encodes a predicted non-specific lipid-transfer protein 3-like protein. The above three genes encoding predicted proteins with complete structural domains and larger molecular weights indicate *Glyma03g04350, Glyma03g04360*, and *Glyma03g04370* maybe the potential candidate genes of *RpsWY* (Fig. 4). A non-specific lipid-transfer protein encoded by *Glyma03g04380* with lower molecular weight and small size protein indicates that *Glyma03g04380* may not be the major potential candidate gene of *RpsWY* (Fig. 4).

## Discussion

There are large numbers of soybean accessions in China, and many PRR resistance germplasm were identified in the previous study (Lohnes et al. 1996; Kyle et al. 1998; Zhu et al. 2006; Sun et al. 2008; Tang et al. 2010; Xia et al. 2011; Ren et al. 2012; Cheng et al. 2015). In our previous investigation, availability of multiple PRR-resistance accessions, including Wayao in South China had been identified (Cheng et al. 2015). Wayao is a PRR-resistance cultivar to *P. sojae* Pm14, Pm28, PNJ1 and P6497 (Table 1). It had a broad-specturm resistance to PRR races or isolates.

In the present study, *RpsWY* is flanked by Satt631 (2.1 cM) and Satt152 (3.9 cM), Satt009 (5.0 cM), Sat_084 (12.7 cM) on chromosome 3 using SSR marker analysis, and mapped to *Rps* gene rich regions on soybean chromosome 3. The results of SNP markers analysis indicated that *RpsWY* gene is located within the interval from 4,466,230 to 4,502,773 bp (36.5 kb) on chromosome 3 falling in the area of the *Rps1k* interval from 4,457,810 to 4,641,921 bp (Gao and Bhattacharyya 2008). Twelve known *Rps* genes were identified and mapped to chromosome 3 before *RpsWY*, including five alleles of *Rps1* (Bernard et al. 1957; Mueller et al. 1978; Anderson and Buzzell 1992), *Rps7* (Weng et al. 2001), *Rps9* (Wu et al. 2011a), *RpsYu25* (Sun et al. 2011), *RpsYD25* (Fan et al. 2009), *RpsYD29* (Zhang et al. 2013a), *RpsUN1*(Lin et al. 2013), a *Rps* gene in Waseshiroge (Sugimoto et al. 2011) and a *Rps* gene in soybean E00003 within the *Rps1k* interval (Zhang et al. 2014). *RpsWY* is a distinct gene from the *Rps1* alleles, because five differentials carrying *Rps1* (*1a, 1b, 1c, 1d*, and *1k*) were PRR-susceptible to *P. sojae* Pm14 in our current study. The position information below SSR marker Satt152 (Fig.S5) suggests that *RpsWY* is distinct from *RpsUN1, RpsYD29* and *Rps7* (Weng et al. 2001; Lin et al. 2013; Zhang et al. 2013a). Otherwise, *Rps9* was flanked by Satt152 (10.1 cM) and Satt631 (7.5 cM) (Fig.S5) (Wu et al. 2011a). *RpsYu25* is located between 3,338,620 and 3,465,436 bp by converting the flanking markers of Satt152 and Sat_186 from the interval 30.1 to 32.8 cM (Fig.S5) (Song et al. 2010; Sun et al. 2011). The locus analysis of SSR markers and sequence interval indicates that *RpsWY* is a distinct gene from *Rps9* and *RpsYu25*. In addition, the *Rps* gene in Waseshiroge is located between Satt009 and T003044871 and may reside in the nucleotide region between 3,919,203 and 4,486,048 (567 kb) on chromosome 3 (Sugimoto et al. 2011). The *Rps* gene in E00003 was located within interval 4,475,877–4,563,799 bp (87.9 kb) on chromosome 3 (Zhang et al. 2014). *RpsWY* and the *Rps* gene from Waseshiroge or E00003 may be allelic, but not *Rps1k*. Therefore, *RpsWY* could be a new allele locus or a new gene. To confirm this finding, additional crosses between Wayao and Waseshiroge or E00003 should be made and genetically analyzed. In previous studies, more than 20 *Rps* genes/alleles have been mapped and designated to ten loci on four soybean chromosomes (13, 18, 16, and 3) since *Rps1* was identified for the first *P. sojae* resistance genes (Lin et al. 2013; Zhang et al. 2014). However, none of them was finely mapped by using low density genetic maps. *Rps1* was one of the *Rps* genes which has been mapped to chromosome 3 of the USDA-ARS soybean Williams isolines (containing the genes *Rj2 Rmd-c Rps2 Ti-a*) approximately 2 cM from locus A071-1 by the restriction fragment length polymorphism (Polzin et al. 1994). Two *Rps1*-k genes were predicted by the sequence analysis using a shotgun sequencing strategy applied in sequencing the BAC contig (Gao et al. 2005). However, more *Rps* genes were mapped on different chromosomes using F_2_, F_2:3_ and/or RIL populations by SSR markers (Demirbas et al. 2001; Weng et al. 2001; Fan et al. 2009; Sugimoto et al. 2011; Sun et al. 2011; Wu et al. 2011a; Lin et al. 2013; Zhang et al. 2013b, c, 2014). In our study, *RpsWY* was mapped on chromosome 3 with 191 F_2_ individuals, which was located in the *Rps*-rich region, by genetic and physical analysis (Fig. S5). The candidate gene of *RpsWY* was further investigated using the 196 RIL population. Fortunately, we found that *RpsWY* was located within interval 4,466,230–4,502,773 bp (36.5 kb) on chromosome 3 covering the sites of four potential candidate genes of *RpsWY*. Analyzing the overlapping region of line 71 and line 100 indicates that the discovery of recombinant monoclonal line 71 and line 100 was crucial for the fine mapping of the candidate genes (Fig. 4).

All the results suggest that high-throughput genome-wide resequencing is an effective method to finely map PRR candidate genes and to help expound their mechanism of disease resistance.

## Electronic supplementary material

Below is the link to the electronic supplementary material.

Supplementary material 1 (DOCX 1689 KB)

## Abbreviations

BSA: Bulk segregation analysis
CTAB: Cetyl trimethylammonium bromide
CIM: Composite interval mapping method
cv.: Cultivar
LOD: Log-likelihood
MSG: Multiplexed shotgun genotyping
PPR: *Phytophthora* root rot
*P. sojae*: *Phytophthora sojae*
QTL: Quantitative trait loci
RIL: Recombinant inbred line
SLAF-seq: Specific length amplified fragment sequencing
SNP: Single nucleotide polymorphism
Taq I: Thermus aquaticus DNA polymerase I
