# Supplementary material for: Fine mapping of a Phytophthora-resistance gene RpsWY in soybean (Glycine max L.) by high-throughput genome-wide sequencing
Source: Theor Appl Genet. 2017 Feb 28;130(5):1041–51. doi: 10.1007/s00122-017-2869-5 (PMC5395582; doi:10.1007/s00122-017-2869-5)
Supplement: Supplementary file 1 — Supplementary material 1 (DOCX 1689 KB) [file 122_2017_2869_MOESM1_ESM.docx]

**SUPPORTING INFORMATION**

**Table S1** The primers’ information of markers linked to resistance gene

| Primers | motifs | Upstream primers | Downstream primers |
| --- | --- | --- | --- |
| Satt152 | (ATT)14 | GCGCTATTCCTATCACAACACA | TAGGGTTGTCACTGTTTTGTTCTTA |
| Satt631 | (ATT)21 | GGTAGATCCAGGAGCTTGAGTCAG | GCGCATCTCACTGCACTTGATTTT |
| Satt009 | (ATT)14 | CCAACTTGAAATTACTAGAGAAA | CTTACTAGCGTATTAACCCTT |
| Sat_084 | (AT)14 | AAAAAAGTATCCATGAAACAA | TTGGGACCTTAGAAGCTA |

The sequences of DNA markers are from the website: <http://www.soybase.org/>

**Table S2** Total numbers of SNPs per chromosome in parents and RILs population

| Chromosome | Total numbers of homozygous SNPs  between parental lines | Total numbers of SNPs  in RILs population |
| --- | --- | --- |
| Gm01 | 63662 | 5425 |
| Gm02 | 38755 | 3280 |
| Gm03 | 37388 | 4471 |
| Gm04 | 57586 | 1108 |
| Gm05 | 18522 | 1473 |
| Gm06 | 39641 | 3697 |
| Gm07 | 30220 | 2738 |
| Gm08 | 30079 | 2517 |
| Gm09 | 30345 | 2877 |
| Gm10 | 10572 | 838 |
| Gm11 | 41272 | 3704 |
| Gm12 | 25833 | 2157 |
| Gm13 | 31056 | 3438 |
| Gm14 | 94719 | 8983 |
| Gm15 | 51909 | 3871 |
| Gm16 | 42470 | 5332 |
| Gm17 | 63883 | 6446 |
| Gm18 | 41644 | 3943 |
| Gm19 | 22296 | 2267 |
| Gm20 | 16030 | 1435 |
| Total | 787882 | 70000 |

**Table S3** The sequencing depth and coverage of the RILs

| sample | coverage | depth（X） | sample | coverage | depth（X） | sample | coverage | depth（X） |
| --- | --- | --- | --- | --- | --- | --- | --- | --- |
| CY-1 | 10.24% | 7.32 | CY-67 | 9.87% | 3.82 | CY-133 | 9.53% | 5.14 |
| CY-2 | 10.48% | 4.77 | CY-68 | 11.34% | 7.77 | CY-134 | 9.97% | 6.46 |
| CY-3 | 7.82% | 2.98 | CY-69 | 10.41% | 3.72 | CY-135 | 9.46% | 5.60 |
| CY-4 | 10.64% | 7.40 | CY-70 | 10.76% | 6.28 | CY-136 | 9.14% | 4.48 |
| CY-5 | 10.54% | 6.85 | CY-71 | 12.22% | 11.73 | CY-137 | 9.49% | 5.05 |
| CY-6 | 10.01% | 6.80 | CY-72 | 11.72% | 9.73 | CY-138 | 9.40% | 5.12 |
| CY-7 | 9.42% | 2.93 | CY-73 | 9.94% | 3.84 | CY-139 | 10.39% | 6.43 |
| CY-8 | 11.20% | 6.14 | CY-74 | 8.14% | 2.83 | CY-140 | 9.28% | 5.15 |
| CY-9 | 10.86% | 7.75 | CY-75 | 4.71% | 1.82 | CY-141 | 9.57% | 5.04 |
| CY-10 | 9.39% | 5.28 | CY-76 | 10.01% | 3.54 | CY-142 | 9.69% | 4.40 |
| CY-11 | 10.06% | 5.03 | CY-77 | 9.04% | 3.78 | CY-143 | 9.07% | 5.29 |
| CY-12 | 7.74% | 3.24 | CY-78 | 10.02% | 4.01 | CY-144 | 10.77% | 9.15 |
| CY-13 | 9.34% | 3.65 | CY-79 | 11.05% | 5.05 | CY-145 | 10.29% | 7.14 |
| CY-14 | 9.55% | 5.52 | CY-80 | 9.52% | 3.84 | CY-146 | 9.49% | 3.65 |
| CY-15 | 10.00% | 4.29 | CY-81 | 11.61% | 5.08 | CY-147 | 7.80% | 2.85 |
| CY-16 | 7.79% | 3.18 | CY-82 | 8.92% | 3.57 | CY-148 | 4.95% | 1.89 |
| CY-17 | 8.08% | 3.54 | CY-83 | 9.88% | 4.04 | CY-149 | 9.70% | 4.58 |
| CY-18 | 10.30% | 4.19 | CY-84 | 7.19% | 2.51 | CY-150 | 9.52% | 4.10 |
| CY-19 | 8.73% | 2.61 | CY-85 | 7.77% | 3.00 | CY-151 | 8.82% | 3.70 |
| CY-20 | 9.10% | 4.58 | CY-86 | 10.42% | 5.47 | CY-152 | 11.24% | 5.65 |
| CY-21 | 9.20% | 5.20 | CY-87 | 10.42% | 5.65 | CY-153 | 8.65% | 3.59 |
| CY-22 | 10.35% | 4.61 | CY-88 | 8.69% | 3.03 | CY-154 | 10.16% | 3.94 |
| CY-23 | 11.11% | 9.98 | CY-89 | 9.83% | 4.71 | CY-155 | 8.51% | 3.23 |
| CY-24 | 10.92% | 7.31 | CY-90 | 10.24% | 3.97 | CY-156 | 9.92% | 4.62 |
| CY-25 | 10.96% | 9.77 | CY-91 | 11.64% | 8.68 | CY-157 | 6.33% | 2.13 |
| CY-26 | 9.36% | 5.04 | CY-92 | 10.84% | 5.78 | CY-158 | 10.29% | 3.32 |
| CY-27 | 8.52% | 4.42 | CY-93 | 12.07% | 4.37 | CY-159 | 10.51% | 5.53 |
| CY-28 | 12.34% | 6.76 | CY-94 | 10.59% | 5.54 | CY-160 | 10.84% | 5.09 |
| CY-29 | 10.74% | 9.13 | CY-95 | 12.42% | 7.03 | CY-161 | 7.67% | 2.27 |
| CY-30 | 10.56% | 8.90 | CY-96 | 11.87% | 6.90 | CY-162 | 10.76% | 3.69 |
| CY-31 | 9.99% | 7.81 | CY-97 | 9.82% | 4.15 | CY-163 | 7.77% | 3.09 |
| CY-32 | 11.41% | 5.73 | CY-98 | 8.65% | 3.45 | CY-164 | 8.47% | 2.69 |
| CY-33 | 12.11% | 7.01 | CY-99 | 5.42% | 2.24 | CY-165 | 9.50% | 4.51 |
| CY-34 | 11.65% | 5.95 | CY-100 | 9.80% | 4.35 | CY-166 | 10.83% | 4.87 |
| CY-35 | 11.07% | 8.29 | CY-101 | 9.62% | 3.29 | CY-167 | 9.86% | 4.16 |
| CY-36 | 8.21% | 3.52 | CY-102 | 9.83% | 3.15 | CY-168 | 10.68% | 5.78 |
| CY-37 | 9.23% | 4.48 | CY-103 | 9.73% | 5.08 | CY-169 | 10.47% | 5.48 |
| CY-38 | 10.77% | 9.68 | CY-104 | 6.85% | 2.50 | CY-170 | 8.48% | 3.44 |
| CY-39 | 10.41% | 7.83 | CY-105 | 10.70% | 4.67 | CY-171 | 7.39% | 2.62 |
| CY-40 | 7.58% | 2.89 | CY-106 | 9.52% | 4.35 | CY-172 | 5.31% | 2.22 |
| CY-41 | 11.33% | 6.80 | CY-107 | 10.61% | 5.02 | CY-173 | 8.86% | 3.17 |
| CY-42 | 6.14% | 2.58 | CY-108 | 6.02% | 2.15 | CY-174 | 8.69% | 4.10 |
| CY-43 | 9.21% | 3.30 | CY-109 | 8.35% | 3.21 | CY-175 | 8.01% | 4.15 |
| CY-44 | 9.78% | 4.33 | CY-110 | 10.77% | 5.86 | CY-176 | 0.30% | 0.01 |
| CY-45 | 10.01% | 4.65 | CY-111 | 10.54% | 5.42 | CY-177 | 8.43% | 3.18 |
| CY-46 | 3.42% | 1.62 | CY-112 | 8.06% | 3.30 | CY-178 | 10.08% | 5.91 |
| CY-47 | 11.13% | 4.77 | CY-113 | 9.94% | 4.58 | CY-179 | 8.58% | 4.43 |
| CY-48 | 9.36% | 4.83 | CY-114 | 8.46% | 3.46 | CY-180 | 8.36% | 4.74 |
| CY-49 | 9.75% | 5.13 | CY-115 | 10.06% | 7.55 | CY-181 | 6.56% | 2.42 |
| CY-50 | 7.00% | 2.30 | CY-116 | 9.85% | 4.66 | CY-182 | 7.93% | 4.00 |
| CY-51 | 6.38% | 2.39 | CY-117 | 9.82% | 6.63 | CY-183 | 8.98% | 5.53 |
| CY-52 | 10.82% | 6.72 | CY-118 | 10.05% | 4.75 | CY-184 | 9.34% | 6.50 |
| CY-53 | 10.98% | 7.47 | CY-119 | 11.75% | 7.37 | CY-185 | 8.65% | 4.61 |
| CY-54 | 9.64% | 4.26 | CY-120 | 11.25% | 4.68 | CY-186 | 8.50% | 4.41 |
| CY-55 | 10.27% | 3.90 | CY-121 | 9.60% | 4.81 | CY-187 | 7.80% | 3.77 |
| CY-56 | 11.25% | 7.25 | CY-122 | 8.99% | 4.64 | CY-188 | 9.14% | 4.21 |
| CY-57 | 10.51% | 6.56 | CY-123 | 5.00% | 1.99 | CY-189 | 9.76% | 5.67 |
| CY-58 | 8.13% | 3.05 | CY-124 | 9.33% | 4.98 | CY-190 | 8.26% | 3.89 |
| CY-59 | 10.41% | 4.78 | CY-125 | 8.25% | 3.65 | CY-191 | 9.50% | 6.41 |
| CY-60 | 8.60% | 3.32 | CY-126 | 9.10% | 4.79 | CY-192 | 10.47% | 7.52 |
| CY-61 | 9.40% | 4.32 | CY-127 | 10.46% | 6.03 | CY-193 | 11.32% | 8.91 |
| CY-62 | 12.00% | 7.78 | CY-128 | 8.52% | 3.95 | CY-194 | 11.01% | 7.35 |
| CY-63 | 11.49% | 7.40 | CY-129 | 9.82% | 6.02 | CY-195 | 5.97% | 2.19 |
| CY-64 | 11.58% | 6.21 | CY-130 | 7.93% | 3.50 | CY-196 | 7.38% | 3.32 |
| CY-65 | 11.46% | 8.60 | CY-131 | 9.20% | 4.89 |  |  |  |
| CY-66 | 10.17% | 5.02 | CY-132 | 5.57% | 2.27 |  |  |  |

**
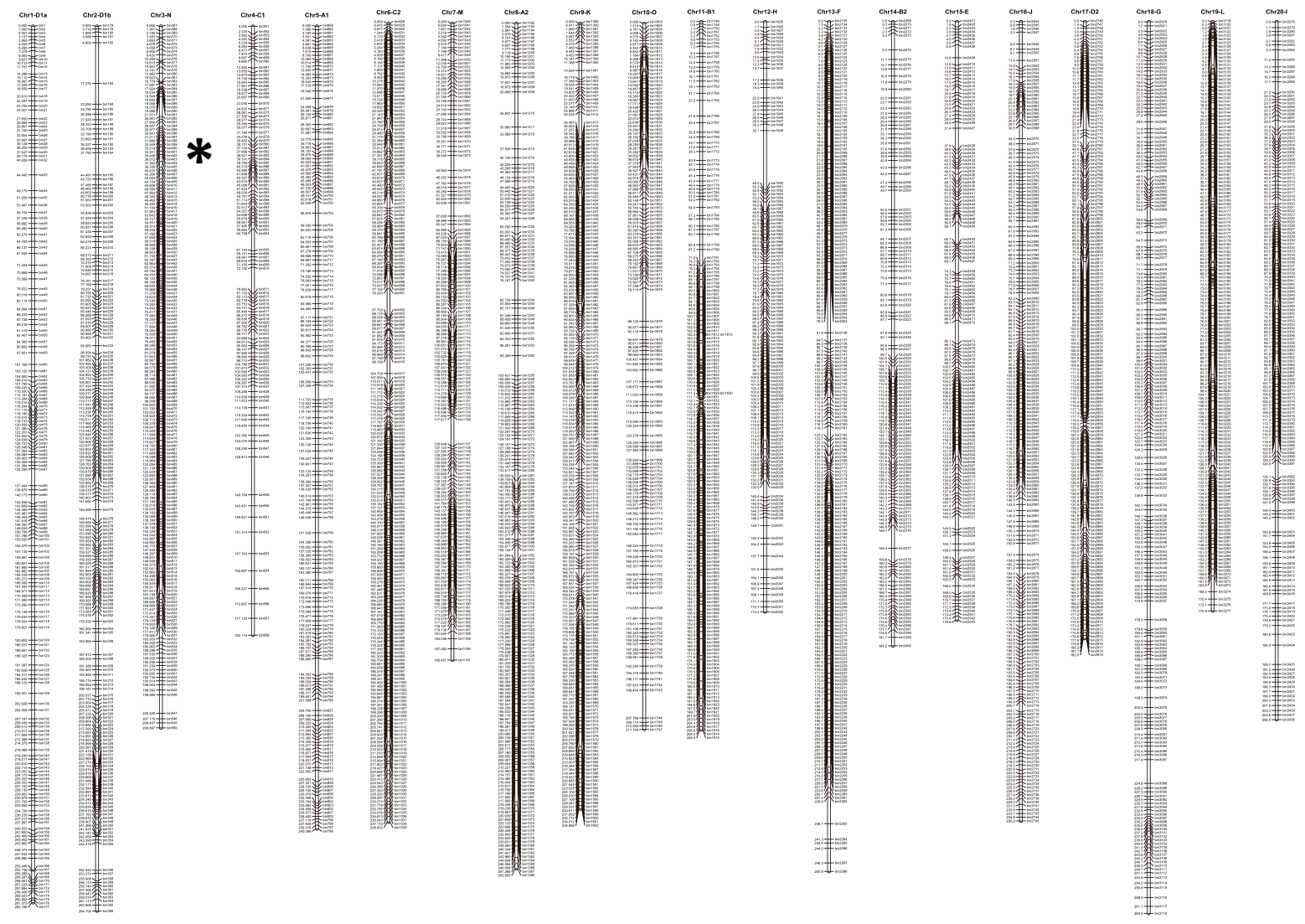
**

**Figure S1** Genetic map of soybean chromosomes

The asterisk indicates the location of bin401.


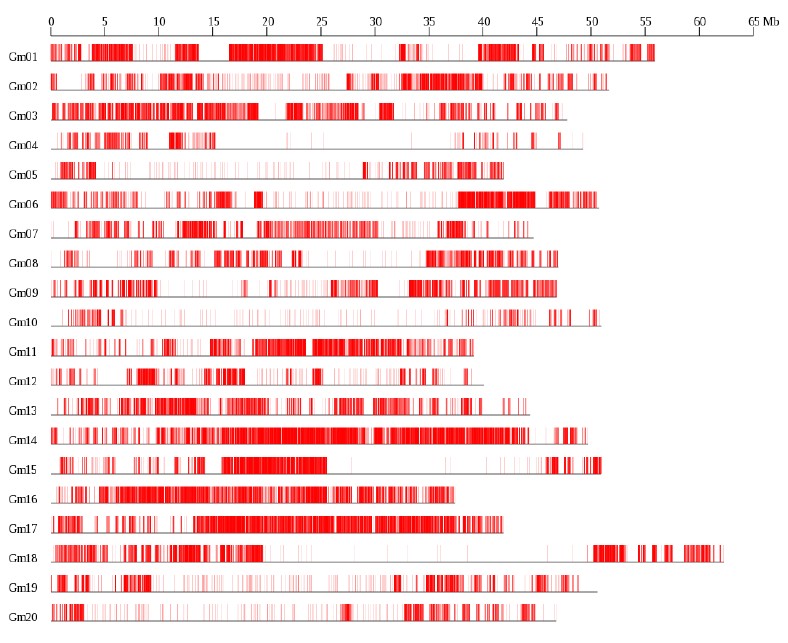


**Figure S2** SNP distribution on the 20 soybean chromosomes


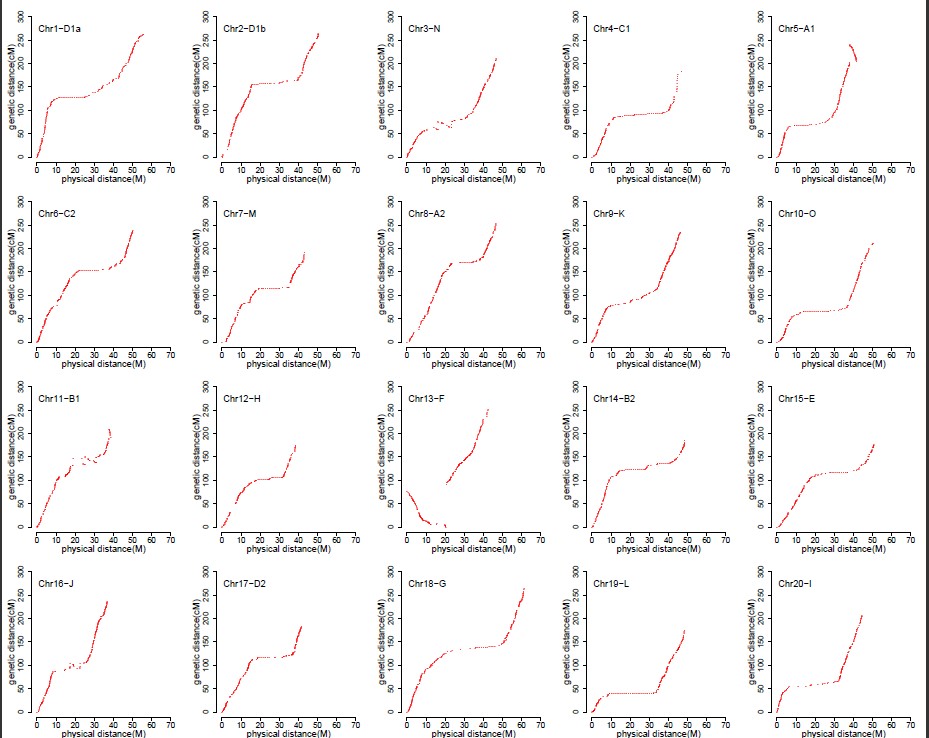


**Figure S3** The linear analysis of genetic map and physical map


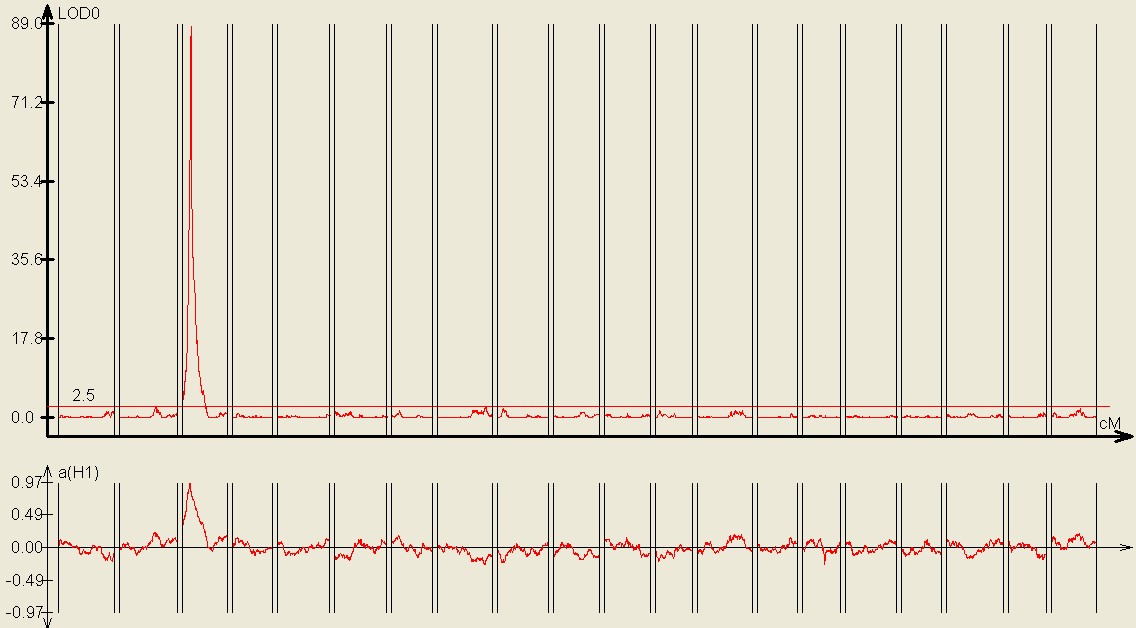


**Figure S4** Gene mapping of the race resistant to *P. sojae* Pm14


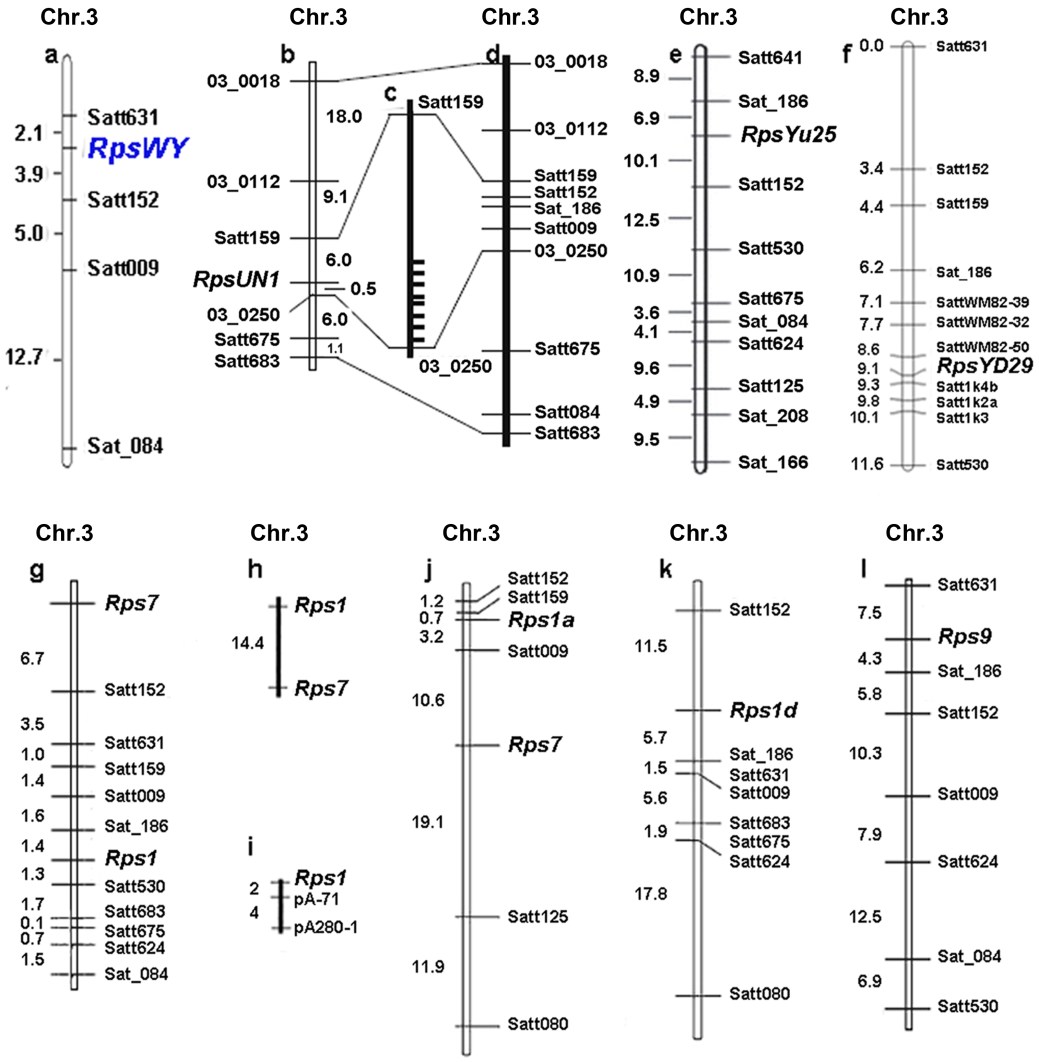


**Figure S5** Genetic and physical map of *RpsWY* and linked *Rps* genes on chromosome 3

**a** Genetic linkage map of *RpsWY* from this study. **b** Linkage map of *RpsUN1* reported by Lin et al (2013). **c** Distribution of annotated NBS-LRR genes within mapped region of *RpsUN1*(Lin et al. 2013). Each bar represents the position of a NBS-LRR gene (Lin et al. 2013). **d** Physical position of SSR markers on chromosome 3 reported by Song et al(2010). **e** Linkage map of *RpsYu25* reported by Sun et al (2011). **f** Linkage map of *RpsYD29* reported by zhang et al (2013a). **g** Composite genetic map of *Rps1* and *Rps7* region reported by Cregan (2003). **h** Linkage map of *Rps1* and *Rps7* reported by Anderson and Buzzell (1992). **i** Linkage map of *Rps1* reported by Diers (1992). **j** Linkage map of *Rps1a* and *Rps*7 reported by Weng et al (2001). **k** Linkage map of *Rps1d* reported by Sugimoto et al (2008). **l** Linkage map of *Rps9* reported by Wu et al(2011a). Marker names and genetic distances (cM) are on the left and right, respectively. Chr.3: chromosome 3.
